# A Long‐Term Clearing Cranial Window for Longitudinal Imaging of Cortical and Calvarial Ischemic Injury through the Intact Skull

**DOI:** 10.1002/advs.202105893

**Published:** 2022-04-09

**Authors:** Chao Zhang, Chun‐Jie Liu, Wei Feng

**Affiliations:** ^1^ Zhanjiang Institute of Clinical Medicine Central People's Hospital of Zhanjiang Zhanjiang Guangdong 524045 China; ^2^ Zhanjiang Central Hospital Guangdong Medical University Zhanjiang Guangdong 524045 China; ^3^ Center for Computational and Genomic Medicine The Children's Hospital of Philadelphia Philadelphia PA 19104 USA

**Keywords:** brain, in vivo optical imaging, ischemic injury, optical clearing, skull

## Abstract

Skull is a reservoir for supplying immune cells that mediate brain immune surveillance. However, during intravital optical imaging of brain, conventional cranial windows requiring skull thinning or removal disrupt brain immunity integrity. Here, a novel long‐term clearing cranial window (LCCW) based on the intact skull, dedicated to chronic skull transparency maintenance, is proposed. It significantly improves optical imaging resolution and depth, by which the cortical and calvarial vascular injury and regeneration processes after ischemic injury are longitudinally monitored in awake mice. Results show that calvarial blood vessels recover earlier than the cortex. And the transcriptome analysis reveals that gene expression patterns and immune cells abundances exist substantial differences between brain and skull after ischemic injury, which may be one of the causes for the time lag between their vascular recovery. These findings bring great enlightenment to vascular regeneration and reconstruction. Moreover, LCCW provides a minimally invasive approach for imaging the brain and skull bone marrow.

## Graphical abstract

To circumvent the major limitations of open‐ and thinned‐skull window, a long‐term clearing cranial window (LCCW) based on the intact skull is developed. LCCW can maintain the skull transparency chronically with single operation, and allows observing the cortical and calvarial microenvironment with high resolution in awake mice, providing a user‐friendly approach for brain science researchers.

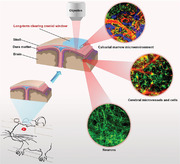

## 1 Introduction

The vasculature is the primary conduit for transporting oxygen, nutrients, cells, etc., thus, it plays an important role in tissue and organ development, regeneration, and remodeling. The cerebrovascular embolization deprives blood supply, resulting in neural dysfunction and brain damage. Many studies have demonstrated that the brain function recovery is highly related to cerebrovascular reperfusion.^[^ ^1^ ^]^ Thus, chronic monitoring of the changes in cerebral vascular structure and function after ischemic injury with high resolution is very important for prognosis assessment and development of neurorehabilitation strategies.^[^ ^2^ ^]^ Additionally, recent studies have found skull bone marrow are myeloid cells and B cells reservoirs for the meninges, and the rich vascular channels serve as pathways for cell migration.^[^ ^3^, ^4^ ^]^ These findings challenge the widely accepted idea that meningeal adaptive immunity originates exclusively from systemic circulation.^[^ ^5^ ^]^ Thus, we envision that calvarial microvessels with a unique microenvironment may have a particular vascular remodeling mode that differs from cerebral vessels, which will bring great enlightenment to vascular regeneration and reconstruction after brain injury.

As powerful tools, optical imaging techniques have been widely used because of their high spatiotemporal resolutions. Examples such as optical coherence tomography (OCT),^[^ ^6^, ^7^ ^]^ photoacoustic imaging,^[^ ^8^ ^]^ and multiphoton laser scanning microscopy imaging^[^ ^9^, ^10^ ^]^ facilitate studies on the structural and functional dynamics of cells and vasculature. However, the imaging depth and resolution are limited due to the high scattering of skull. Therefore, various cranial windows have been developed in recent decades, mainly including thinned skull window,^[^ ^11^ ^]^ open‐skull glass window,^[^ ^12^, ^13^ ^]^ and some other implanted windows.^[^ ^14^, ^15^, ^16^ ^]^ Although these cranial window modalities based on delicate surgeries enables comprehensive investigations of brain structure and function in neuroscience research, they still have some disadvantages.^[^ ^17^ ^]^ Recent studies demonstrate that meninges contain a pool of monocytes, neutrophils, and B cells supplied by the adjacent skull and vertebral bone marrow rather than the blood,^[^ ^4^, ^5^ ^]^ which means that the skull is an important part of the brain immunity. The skull removal or polish during their establishment will disrupt the skull integrity, which may cause misunderstanding to the real physiopathological process. Therefore, it is of great significance to establish a cranial window based on the intact skull. In recent years, several in vivo optical clearing skull windows have been proposed,^[^ ^18^, ^19^ ^]^ which are realized by topical application of various skull optical clearing agents, allowing to observe cortical vessels and cells with high resolution. However, current optical clearing skull windows are temporary and need to be established each time in chronic studies, which is time‐consuming and complex. Moreover, these optical clearing windows are not practical on awake mice.

To address these problems, we develop a novel long‐term clearing cranial window (LCCW) based on the intact skull, dedicated to maintaining the skull transparency chronically, avoiding repeated clearing treatments, and improving animal welfare. We introduce the detailed procedure to establish LCCW. The improvement of imaging depth and resolution leads to the clear observation of the cortical/calvarial blood vessels, neurons, and microglia/monocytes. The skull transparency can maintain for at least two months with single operation, allowing to continuously trace the physiopathological process of brain and skull. We longitudinally monitor the vascular dynamic changes of cortex and skull in awake mice after the ischemic injury and explore the changes in the gene expression profile at specific time points. The accomplishment of this research not only provides a well‐performed skull window for intravital brain studies, but also has great value in developing therapy strategies for tissue ischemic injury.

## 2 Results

### 2.1 The Establishment of LCCW

To overcome the limitations from the existing cranial windows, we propose a novel method named long‐term clearing cranial window (LCCW).

**Figure 1:**
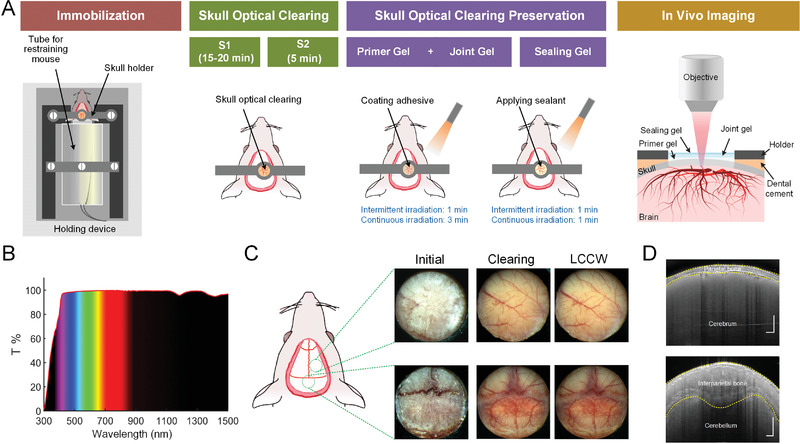
The establishment of LCCW. A) Schematic representation of LCCW establishment procedure. B) Transmittance through solid skull clearing maintenance reagent relative to blank cover glass. C) Representative photograph of dry skull (Initial), optical clearing window (Clearing), and LCCW in eight‐week‐old mice. D) OCT cross‐sectional images. Skull and cortex are labeled from the top to the bottom, and the boundaries between air and tissue, as well as skull and brain are outlined by yellow dotted curves (Scale bar: 250 µm).

The establishment of the LCCW includes four steps. First, the mice experienced scalp removal, mucosa cleanup, and holder adhesion is immobilized in a customer‐built holding device (**Figure** **1**A and Figure S1, Supporting Information). Second, we add clearing solution S_1_ to the skull, and rub the skull gently with a swab for accelerating the clearing speed. About 15 min later, the solution S_2_ is applied to the skull and subsequently removed after the skull becomes transparent. Third, to maintain the skull transparency chronically, we add the skull clearing maintenance reagents that are composed of primer gel, joint gel and sealing gel. A spot of primer gel is smeared on the skull, followed by a drop of joint gel mixed uniformly. Next, we use a LED light to irradiate at a distance of 30 cm for about 4 min to let it fully solidify. In this process, the mixed gel is intermittently irradiated in the first 1 min with the cycle of 3 s irradiation and 3 s nonirradiation. Then, it undergoes continuous irradiation for another 3 min. Finally, a layer of sealing gel is applied and solidified by the same LED light in the periodic irradiation pattern for 1 min, followed by continuous irradiation for another 1 min.

In addition, the transmittance of solid skull clearing maintenance reagent is measured from 300 to 1500 nm (Figure 1B). The result shows that the light penetration is high in the range between 400 and 1500 nm, especially at the waveband of 400–1100 nm and 1200–1300 nm, which is nearly up to 100%. And, we obtain the direct viewings of cerebral and calvarial blood vessels under different conditions (Figure 1C). The result shows that neither the cortical nor calvarial blood vessels are visible at the initial state. Only with the usage of skull optical clearing solutions can we observe the vessels. Furthermore, by applying skull clearing maintenance reagent, the skull clearing state is excellently preserved, permitting us to monitor the cerebral and calvarial blood vessels in a long term. And Figure 1D presents that the heterogeneous interparietal bone is much thicker than parietal bone, which contains more abundant monocytes and vessels (Figure S2, Supporting Information), hence, it is a very suitable site for cranial bone marrow imaging.

### 2.2 Imaging Performance of LCCW

**Figure 2:**
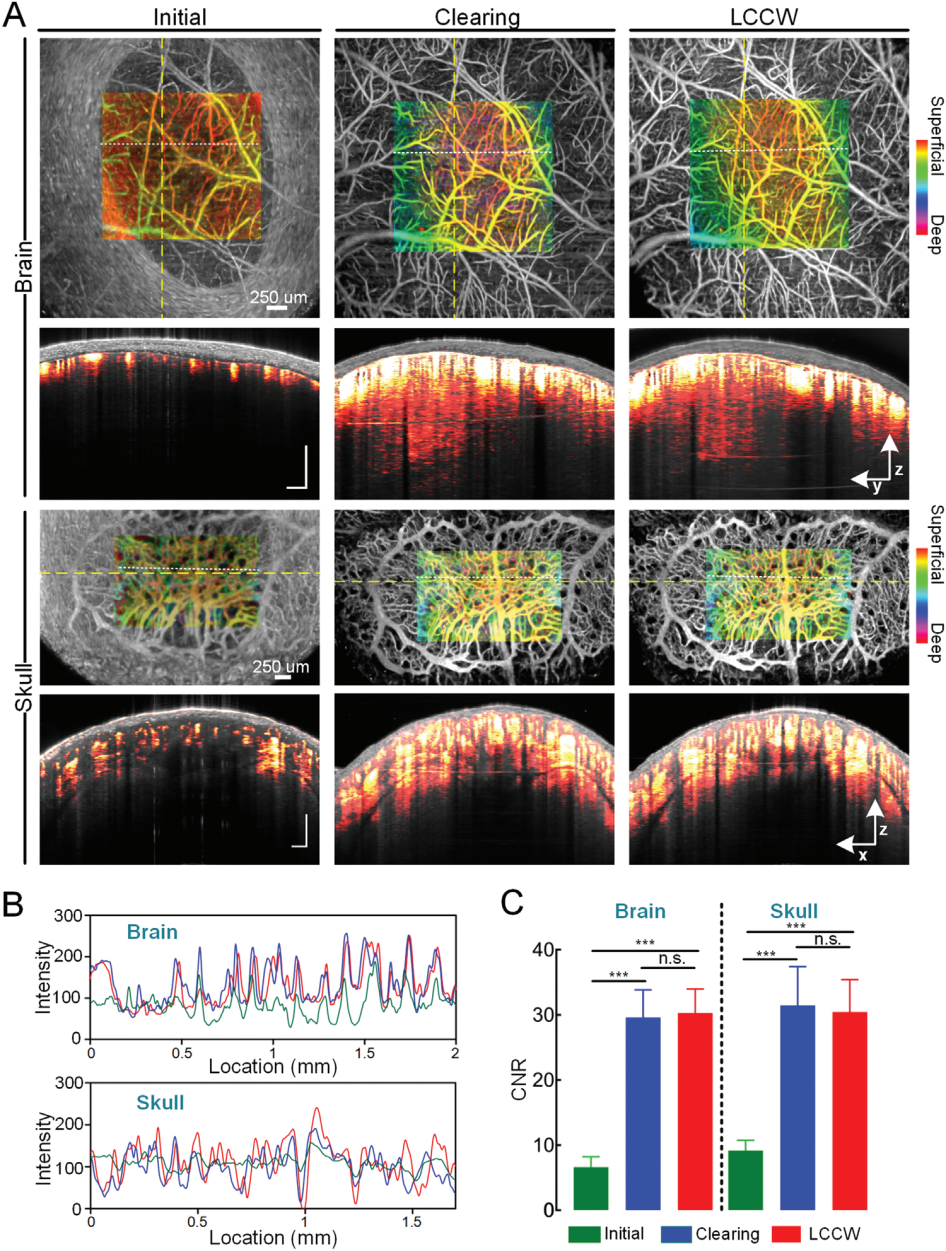
The imaging performance of LCCW on OCT. A) Imaging blood vessels through dry skull (Initial), optical clearing window (Clearing), and LCCW in eight‐week‐old mice. The maps are vascular maximum projection (MIP) view in depth direction (from surface to depth 560 µm). The cross‐sectional angiograms are from yellow dotted lines (Gray: OCT structure; Red hot: blood vessels). B) The profiles of intensity maps along the white dotted line in (A). C) The CNR values of images (*n* = 7, mean ± standard deviation, n.s.: no significance; *p < 0.05; **p < 0.01; ***p < 0.001).

To verify the imaging performance of LCCW, we use 1300 nm‐OCT to analyze the vascular imaging quality in cortex and skull (**Figure** **2**). As shown in Figure 2A, the high scattering of skull largely limits the imaging depth, and the details of many blood vessels in angiographic images are almost indistinguishable. After the treatment with in vivo skull optical clearing solutions, the imaging depth is strongly enhanced, permitting to observe deeper cerebral and calvarial blood vessels with much higher resolution. Furthermore, with the establishment of LCCW, the skull transparency maintains well, and the imaging performance is as good as that in optical clearing state.

The imaging qualities under different conditions are quantified in Figure 2B,C. The results show that the signal peaks of cerebral and calvarial microvessels based on LCCW and optical clearing skull window strongly increase compared to that in the initial condition. As for contrast‐to‐noise ratio (CNR), it also significantly increases after the applications of optical clearing window and LCCW, and no significant difference existed between LCCW and optical clearing window. These results confirm that LCCW can indeed improve the optical imaging performances, which provides a powerful tool for dynamically monitoring the structural and functional changes in cerebral and calvarial blood vessels through the intact skull.

**Figure 3:**
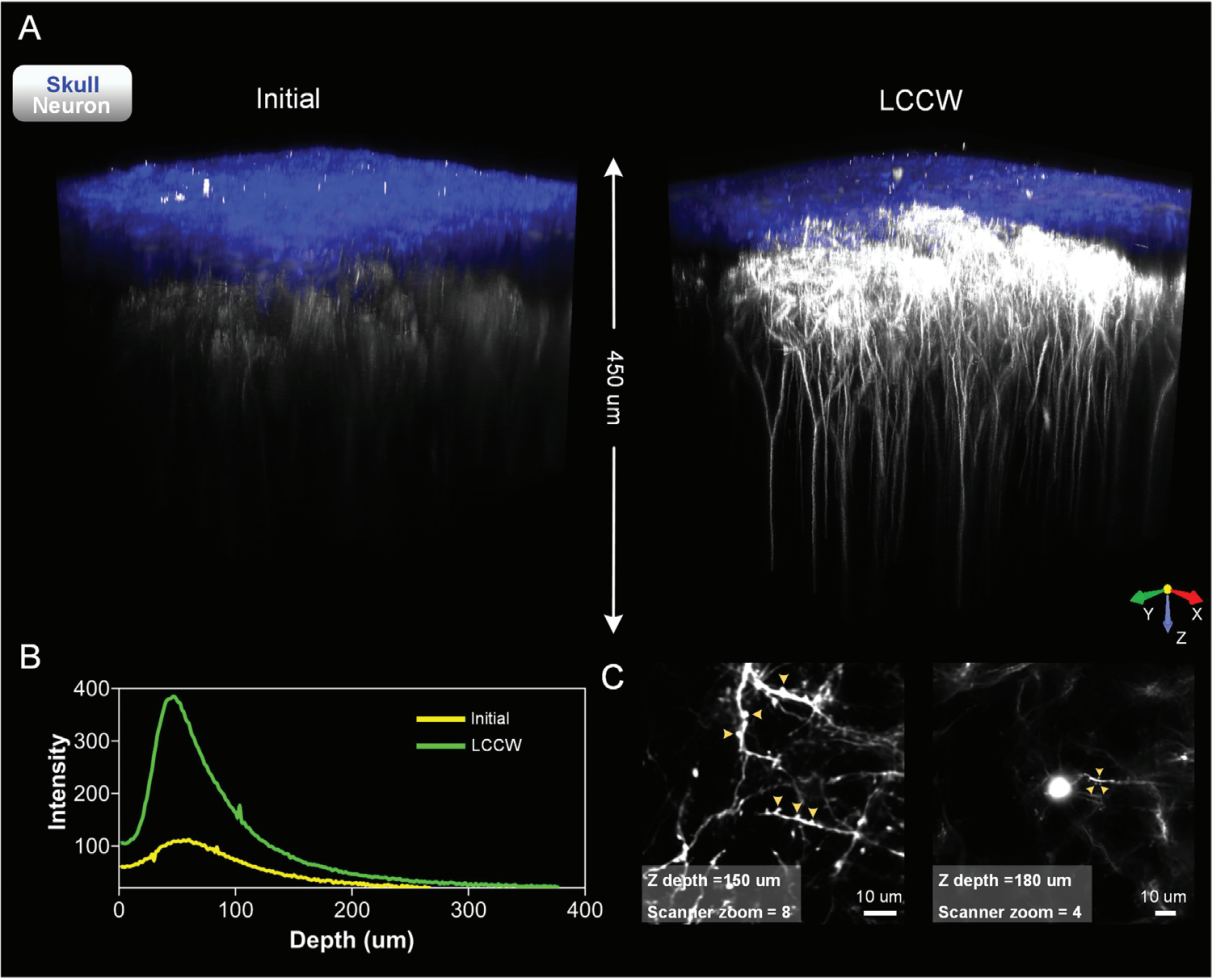
Two‐photon imaging of cortical neurons through LCCW. A) Typical 3D view of neuron in ten‐week‐old *thy1‐YFP* mice before and after the LCCW establishment. B) Depth‐directional profiles of fluorescence signal intensity of neurons. C) Representative images of two‐photon microscopy for synaptic imaging and neural cell body (arrows point to the dendrite spines).

Furthermore, we characterize the imaging performance of LCCW for two‐photon microscopic imaging in **Figure** **3**. With the same imaging parameters, LCCW effectively improves the imaging depth and fluorescence signal intensity (Figure 3A,B). This allows us to clearly distinguish dendrite spines and neural cell body as shown in Figure 3C. Besides, to explore possible optical aberration in microscopic imaging caused by the refractive index mismatch between water and solid skull clearing maintenance reagent, we image 200 nm fluorescent beads under two imaging conditions (Figure S3, Supporting Information). The solid skull clearing maintenance reagent causes minimal effects on both radial and axial resolutions. In summary, LCCW can also be employed in fluorescence microscopic imaging for enhancing the imaging performance.

### 2.3 Long‐Term Monitoring of Cortical and Calvarial Blood Vessels via LCCW

To demonstrate the long‐term imaging stability of LCCW, we monitor the changes of cerebral and calvarial blood vessels in adult mice over a period of 8 weeks using OCT imaging system on awake mice.

**Figure 4:**
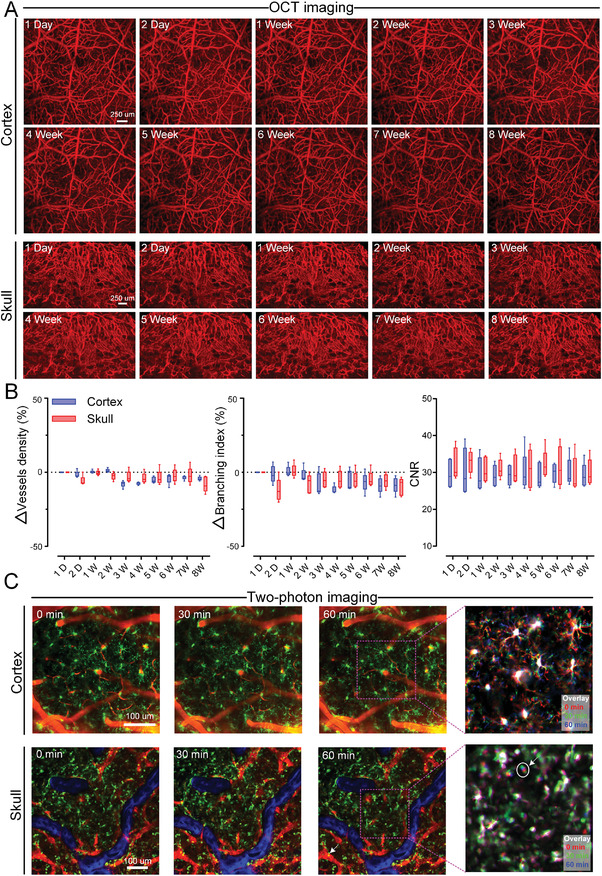
The evaluation of long‐term imaging performance of LCCW for the cortex and skull. A) Representative angiograms of cerebral and calvarial blood vessels at different time points (MIP in depth direction (from surface to depth 560 µm). B) Longitudinal monitoring of the relative changes in vessels density, branching index [(Values_different time_ − Values_1 D_)/Values_1 D_] and the CNR values (*n* = 5, mean ± standard deviation). C) Monitoring the activation of microglia in cortex and monocytes in skull within 1 h (Green: microglia or monocytes; Red: vessel; Blue: skull SHG).

**Figure** **4**A shows vascular MIP maps for each week. We observe that the cerebral and calvarial microvascular distribution patterns and morphology show no obvious change. Later, we quantify the changes in vascular structure, and the results show that the vascular density and branching index of cortex and skull do not fluctuate much (Figure 4B). The CNR values for the indicated period in weeks barely change, indicating that LCCW can excellently maintain the imaging performance for the long term. In addition, we investigate the possible inflammation induced by the establishment of LCCW on the cortex and skull. We observe the migration of microglia in the cortex and monocytes in the skull marrow cavity within one hour in *Cx3cr1^EGFP/+^* mice (Figure 4C). It can be found that the position of microglia in the cortex barely migrates, except for the occasional foot swing. As for the monocytes in skull marrow cavity, most of them hardly migrate over time. However, in some locations, such as the circle indicated by the arrow, the cells have a tiny movement with blood flow. Movie S1 (Supporting Information) presents the movement of monocytes in skull marrow cavity over time. We notice that cells outside the vessels do not have obvious movement, but some particular ones in vessels migrate. In summary, the stability of cortical/calvarial vessels and microglia/monocytes cells shows that the LCCW hardly affects the cortical and cranial microenvironment, thus, it can be a safe tool for intravital imaging.

### 2.4 LCCW Enables Longitudinal Tracing of Cerebral/Calvarial Vascular Dynamics after Ischemic Injury

Noninvasive monitoring of the spatial‐temporal vascular dynamic pattern in awake mice plays a key role in understanding the vascular injury and regeneration processes in natural conditions. Here, we longitudinally monitor the changes of cerebral and calvarial blood vessels in focal photothrombosis (PT) ischemic injury model on awake mice.

**Figure 5:**
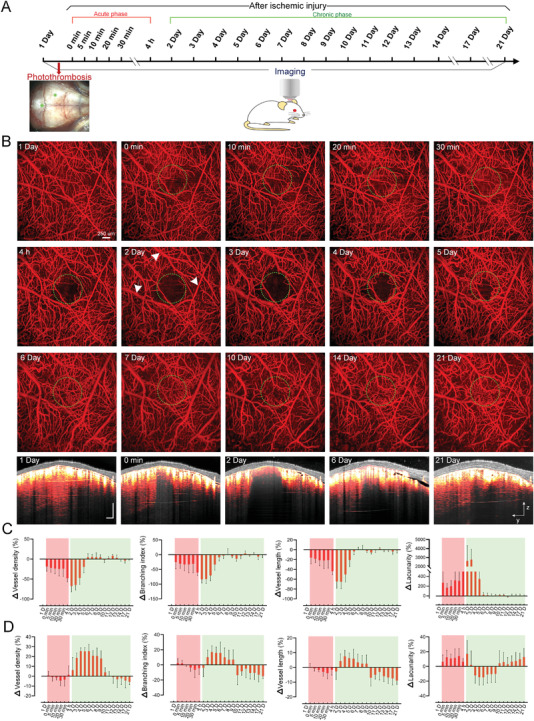
Longitudinal monitoring of cerebral vascular dynamics through LCCW in acute and chronic phases post PT. A) Timeline of longitudinal imaging. The red arrow indicates PT, and the green dots point the irradiation sites where PT is formed. B) Typical MIP views of the cerebral vascular maps at different time points (from surface to depth 560 µm). Green dotted circles represent the irradiation region, and the white arrows indicate representative altered blood vessels. The cross‐sectional angiograms are from the same location at typical time point (Gray: OCT structure; Red hot: blood vessels). C,D) Longitudinal monitoring of the relative changes in vessels density, branching index, vessel length, and lacunarity [(Values_different time_ − Values_1 D_)/Values_1 D_] in and outside the dotted circles, respectively (*n* = 5, mean ± standard deviation, Red shade: acute phase post PT; Green shade: chronic phase post PT).

**Figure** **5**A shows the experimental schedule composed of acute and chronic stages after PT. Figure 5B presents the vascular variations in the core ischemic region and peripheral area in both acute (0–30 min and 4 h) and chronic (2–21 d) phases in cortex. Within 30 min after the laser irradiation, most of the small vessels in the targeted area are blocked completely, in contrast to the large vessels. 4 h later, most blood vessels in the irradiated area disappear. The boundary of the core ischemia area roughly matches the laser irradiation area shown in the green dotted circle, and vessels outside the core ischemic area show negligible change. On the second and third days, the area of ischemia region expands, and the morphology of blood vessels outside the ischemia core also changes (pointed by white arrows in Figure 5B). On the fourth day, the vessels reappear gradually, and the growth almost stops on the sixth day. Thereafter, the vessels in the core ischemic area maintain a dynamic pruning and regenerating status and become stable on the 14th day. We further analyze the cross‐sectional angiograms of the core ischemic area. In the normal state, the OCT signal distributes uniformly. After PT, the bottom signal disappears except for the superficial layer, and the range of OCT signal loss region expands on the second day. On the sixth day, the signal at the upper layer appears to have recovered, but there still are some deficiencies at the bottom, which is similar to that on the 21st day.

We further quantitatively analyze the relative changes of vascular structure with various parameters, including vascular density, branch, length, and lacunarity in and outside the core ischemic area during the acute and chronic stages (Figure 5C,D). For the vessels in the core ischemic area at the acute phase (0 min to 4 h), the variation trends of vascular density, branch, and length are similar. These parameters decrease ≈25% within the first 30 min and further go as low as ≈50% within 4 h. At the chronic phase, these three parameters reach a minimum on the second and third days and then gradually increase. From the sixth day, these parameters almost recover to their initial levels and remain stable. As for the lacunarity, its variation is completely opposite to the other three parameters (Figure 5C). Toward the vessels outside the core ischemic area at the acute phase (0 min to 4 h), the overall trends of vascular density, branch, and length go slightly downward. During the chronic stage, these parameters rise first and then fall, but they are higher than the initial state from the second to the tenth day. From the 11th day, these parameters became lower than normal, resulting from the vessel pruning. For the lacunarity, the variation is also nearly opposite to the other three parameters (Figure 5D).

**Figure 6:**
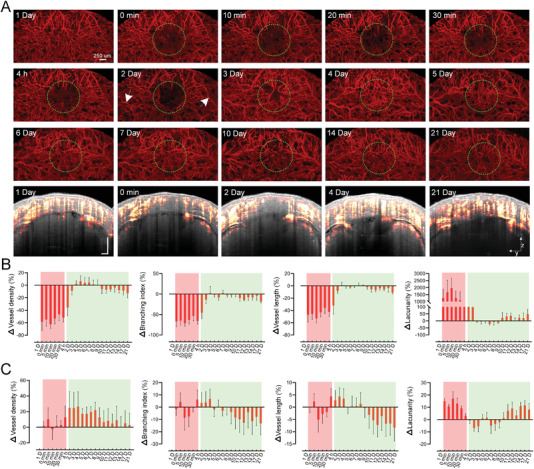
Longitudinal monitoring of calvarial vascular dynamics through LCCW in acute and chronic phases post PT. A) Typical MIP views of the calvarial vascular maps at different time points (from surface to depth 560 µm). Green dotted circles represent the irradiation region, and the white arrows indicate representative altered blood vessels. The cross‐sectional angiograms are from the same location at typical time point (Gray: OCT structure; Red hot: blood vessels). B,C) Longitudinal monitoring of the relative changes in vessels density, branching index, vessel length and lacunarity [(Values_different time_ − Values_1 D_)/Values_1 D_] in and outside the dotted circles, respectively (*n* = 5, mean ± standard deviation, Red shade: acute phase post PT; Green shade: chronic phase post PT).

We also monitor the calvarial vascular changes after PT with the establishment of LCCW on interparietal bone. **Figure** **6**A shows typical MIP views of calvarial blood vessels over the acute and chronic stages post PT. Within 4 h after laser irradiation, the calvarial blood vessels are almost completely blocked in the core ischemic area, and there is no obvious change in the vascular structure outside the ischemic region. From the second day, some new blood vessels appear in the ischemic core area, and the morphology of calvarial blood vessels outside the ischemic area changes (pointed by white arrows in Figure 6A). On the fourth day, the calvarial vascular perfusion appears to recover completely. As the cross‐sectional angiograms in the core ischemic area show, the vascular signal at the bottom disappears except for that from the superficial after PT. The vascular signal loss region expands on the second day. On the fourth day, the signal appears to be recovered, which is similar to that on the 21st day.

The relative changes of vascular parameters are also quantitatively analyzed during the acute and chronic stages post PT in and outside the core ischemic area (Figure 6C,D). Different from the changes in cerebral vessels, at acute phase (0 min to 4 h), the calvarial vascular parameters, including vascular density, branch, and length in the ischemic area, almost reach the minimum at the moment of PT‐0 min. At the chronic phase (2nd day to 21st day), these three parameters gradually increase and almost recover to the normal level on the fourth day, and then fluctuate in the following days. Similarly, the variation of lacunarity in the core ischemic area is opposite to the other parameters (Figure 6C). As for the parameters outside the ischemic region, the vascular density is generally higher than the initial level. The branch and length dynamically change at the acute phase (0 min to 4 h) and during the second to the eighth day. At other times, these two parameters remain lower than the initial level. For the lacunarity, its trend is almost opposite to the other two parameters as well.

### 2.5 Differences in Gene Expression Profiles between Brain and Skull after Ischemic Injury

According to the above analysis about cortical and calvarial vascular injury and regeneration patterns after ischemic injury, vascular damage of both the cortex and the skull almost reaches the maximum on the second day. For the brain, regenerated vessels almost cover the entire injured area by the sixth day. However, these occur on the fourth day for the skull. To explore the reasons for the differences in vascular recovery between the brain and the skull after ischemic injury, we perform the transcriptome analysis at these typical time points.

**Figure 7:**
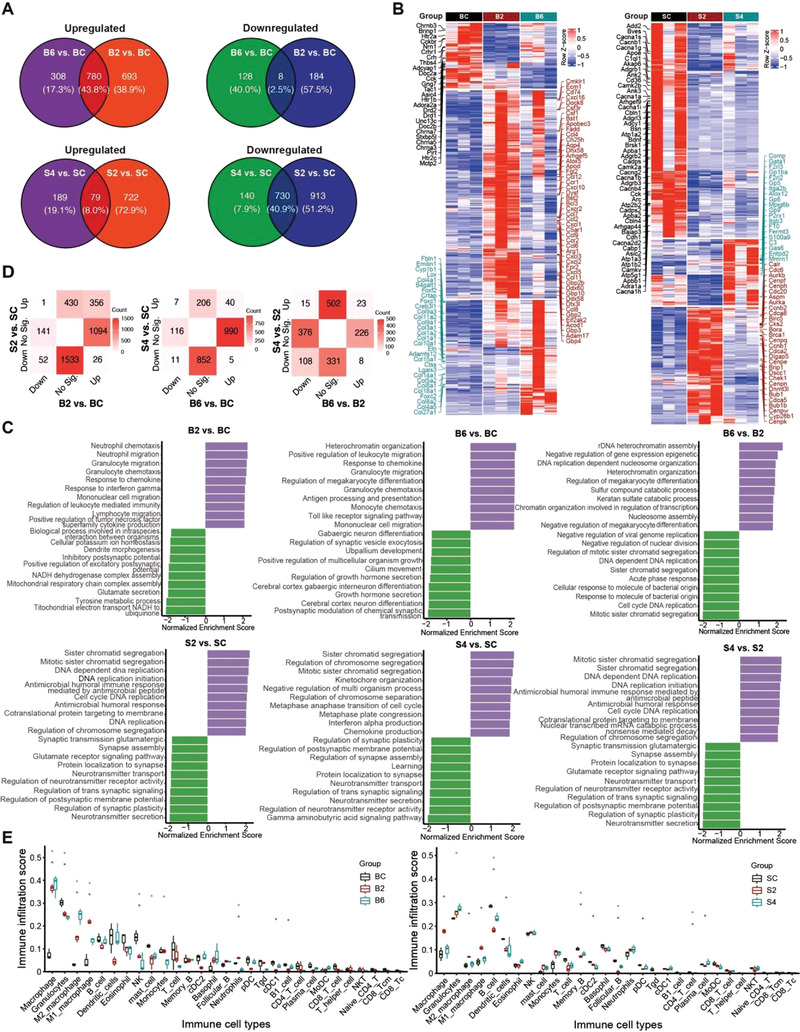
The differences of gene expression profiles between the brain and the skull after ischemic injury. A) Venn diagram for number of up‐ and downregulated genes between brain and skull. B) Heatmap of control and injury samples with DEGs. C) Gene set enrichment analysis (GSEA) for the cortical and calvarial ischemic injury with adjusted *p*‐value less than 0.05 (Top 10 terms). D) Comparative analysis of up‐ and downregulation of DEGs between brain and skull in different timepoints of ischemic injury. Up is FC > 2 and FDR < 0.05, Down is FC < 1/2 and FDR < 0.05. No Sig. is not significant. E) Boxplot of immune cell abundance differences for brain and skull, asterisk is *P*‐value less than 0.05 between control and injured group (BC: control group for uninjured brain; B2/6: brain group for the 2/6 day after ischemic injury; SC: control group for uninjured skull; S2/4: skull group for the 2/6 day after ischemic injury).

We categorize them as shown in PCA plots (Figure S4, Supporting Information). The number of differentially expressed genes (DEG, FDR<0.05 and |log_2_(Fold Change)|>1) in the brain is fewer than that in the skull after ischemic injury (Table S1, Supporting Information). For cerebral ischemic injury on the sixth and the second days (vs control), they share 43.8% upregulated and 2.5% downregulated genes, respectively. For cranial ischemic injury on the fourth and the second day (vs control), the co‐upregulated and co‐downregulated genes are 8.0% and 40.9%, respectively (**Figure** **7**A). The greatest changes in the number of DEGs happen on the second day after ischemic injury both in the cortex and skull, which is consistent with the blood vessels injury patterns.

Furthermore, we investigate the expression patterns of DEGs across the different timepoints after the ischemic injury, as shown in heatmaps (Figure 7B). Hierarchical clustering analysis reveals that the upregulated and downregulated genes are distinguished. And, we identify that the biological processes alter at each time point in each site (Figure S5A, Supporting Information). For the brain, significantly enriched Gene Ontology (GO) terms for downregulated genes on the second and the sixth days include multicellular organismal response to stress, signal release from synapse, neurotransmitter secretion and other biological processes related to neural activity. The upregulated genes on the second day are mainly enriched in the immune response and the inflammatory response biological processes, whereas the genes related to the extracellular matrix organization, collagen fibril organization, and other regulations of the immune system process upregulate on the sixth day. Interestingly, for the skull, the genes associated with synaptic function and neural activity are significantly downregulated on the second and the fourth days. Otherwise, both the genes related to cell division and cell cycle on the second day and the genes related to coagulation, inflammatory response, and wound healing on the fourth day show significant upregulation (Figure S5A, Supporting Information).

Gene Set Enrichment Analysis (GSEA) also shows quantitative differences between the brain and the skull regarding ischemic injury (Figure 7C). On the second day after ischemic injury in the brain (Figure 7C, B2 vs BC), the upregulated biological processes are mostly associated with the immune cell migration, whereas the processes related to cellular component organization, cellular metabolic process, and response to stimulus appear to be downregulated; in the skull (Figure 7C, S2 vs SC), the upregulated biological processes are mostly enriched in cell cycle, cellular component organization, and response to stimulus, while the downregulated biological processes mainly include cell communication and biological regulation. For the sixth day after ischemic injury in the brain (Figure 7C, B6 vs BC), the processes associated with the cellular component organization, response to stimulus, and immune cell migration turn out to be upregulated, while the downregulated biological processes are mostly enriched in cell communication and developmental process; in the skull (Figure 7C, S4 vs SC), the processes related to cellular component organization and cell cycle show upregulation, while most of the biological processes about cell communication show downregulation. When comparing ischemic injury on the sixth day with that on the second day in the cortex (Figure 7C, B6 vs B2), we find that the upregulated processes are mostly associated with the developmental process, whereas the biological processes related to cell cycle, cellular component organization and cellular component organization appear to be downregulated. When comparing the ischemic injury on the fourth day with that on the second day in the skull (Figure 7C, S4 vs S2), we observe upregulation of developmental process but the processes regarding the cellular component organization, response to stimulus, and multicellular organismal reproductive show downregulation.

Then, we perform a quantitative comparison of the DEGs between the brain and the skull after ischemic injury as shown in Figure 7D. On the second day after ischemic injury (vs control), the 1533 genes, mostly related to regulation of synapse and neurotransmitter transport, shows significantly downregulation only in the skull (Figure S6, Supporting Information: GO term enrichment analysis); the 1094 genes, related to the regulation of immune response, show significant upregulation only in the brain; the 430 genes associated with cell division and extracellular matrix organization significantly upregulates only in the skull, which are related to vascular BBB dysfunction. On the sixth and fourth day after ischemic injury (vs control), the 852 genes appear to be significantly downregulated only in the skull, most of which are associated with the regulation of synapse and neurotransmitter transport (Figure S7, Supporting Information: GO analysis); the 116 genes turn to be downregulated only in the brain, which are mainly involved in regulation of neurotransmitter; the 206 genes, associated with coagulation and wound healing, show significant upregulation only in the skull (Figure S7, Supporting Information: GO analysis); the 990 genes, mostly related to regulation of immune response and extracellular matrix organization, show significant upregulation only in the brain. On the sixth and fourth day after ischemic injury (vs second day), the 331 genes become significantly downregulated only in the skull, which are mainly involved in metabolic process, vasculature development, and regulation of angiogenesis (Figure S8, Supporting Information: GO analysis); the 376 genes become significantly downregulated only in the brain, most of which are associated with cell division and cell cycle; the 502 genes become significantly upregulated only in the skull, most of which are related to the activation and proliferation of immune cells (Figure S8, Supporting Information: GO analysis); the 226 genes become upregulated only in the brain, which are mainly involved in extracellular matrix organization and inflammatory response.

Moreover, recruitment of the inflammatory infiltrate plays a key role in angiogenesis and tissue remodeling. The innate and adaptive immune cells are involved in the mechanisms of endothelial cell proliferation, migration, and activation by producing and releasing a large set of pro‐angiogenic mediators. Here, we use ImmuCellAI‐mouse to predict the abundance of immune cells and to reveal the changes of immune cell infiltration during ischemic injury (Figure 7E). We find that the immune cells in the brain fluctuate more dramatically than that in the skull after ischemic injury. Multiple types of immune cells may take part in the process and there seems to be a large difference in changes of some immune cells between the brain and the skull. After the ischemic injury, the abundance of macrophages (M1, M2) in the brain and the skull increases, however, only those in the skull return close to the normal levels by the fourth day. The variation of granulocytes and monocytes abundance in the brain and the skull are almost opposite. Besides, the abundance of B cells and memory B cells in the brain are barely affected but show significant changes in the skull. The abundance of some other immune cells (NK, mast cell, neutrophils, cDC1) has no change in the skull but shows significant changes in the brain. The alterations of immune cell abundances can lead to the changes in immune microenvironments, which might cause the time lag of blood vessels recovery between the brain and the skull after the ischemic injury.

## 3 Discussion

In this work, a novel cranial window, namely LCCW, is developed, featured with high transparency, convenience, long‐term effectiveness, and safety. The LCCW enables us to track the vascular injury and regeneration process in both the cortex and the skull with high spatial‐temporal resolution through the intact skull. Such chronic imaging is hard to realize through the initial turbid skull because of the refractive index mismatch among the complex compositions of the skull. During the past decades, several kinds of cranial windows are proposed to solve this problem, such as open‐skull glass window, thinned‐skull window, and some other derived cranial windows.^[^ ^17^ ^]^ However, there exist many deficiencies among these methods. For example, the removal of the skull not only causes injury to the dura matter but also induces cortical inflammations.^[^ ^20^, ^21^ ^]^ As a result, the tissue remains opaque and inappropriate for imaging for two weeks after the surgery. Thinning the skull makes it fragile and proliferous, thus, it is challenging to perform repeated imaging. Meanwhile, these cranial windows require delicate surgical operations, so the success ratio is limited. Even with successful surgery, the removal and polish of the skull can disturb the immune responses to the brain, leading to some misunderstanding of the real pathophysiological process. This is because recent studies indicate that the bone marrow niches adjacent to the brain supply monocytes and B cells to the meninges in both physiological and pathological conditions, which plays an important role in regulating the immune responses during neurological diseases.^[^ ^4^, ^5^ ^]^ The rapid development of tissue optical clearing techniques in recent years provides us with a new idea,^[^ ^22^, ^23^ ^]^ and a variety of optical clearing skull windows have been proposed.^[^ ^19^, ^24^, ^25^, ^26^ ^]^ These cranial windows allow observing the structural and functional changes in cortical vessels and neurons by combining with various optical imaging techniques. However, the clearing states of the skull cannot be preserved for days, not to mention for months, thus it requires repeated clearing windows establishment in each imaging session. The repeated anesthesia and clearing operations not only waste time but also raise the difficulty and complexity of the experiment. Thus, there is an urgent need to develop a stable window that satisfies the long‐term observation of the cortex with high resolution via a single operation. In this work, we develop such a skull clearing maintenance method and establish the LCCW.

Recently, Li et al. also developed a solid optical clearing agent for long‐term monitoring of cortex.^[^ ^27^ ^]^ Compared with the results they currently show, our LCCW maintains the skull transparency for even longer time. Besides, the LCCW is applicable for both the parietal and interparietal bone, which enables us to perform the longitudinal imaging of cortical and calvarial vascular regeneration process after ischemic injuries. As for their method, the effectiveness on interparietal bone remains to be assessed. For the LCCW, the solidified skull transparency maintenance reagent has proper hardness and flexibility, as shown in Movie S2 (Supporting Information). Moreover, the LCCW is sealed by the tape during nonimaging periods, so as to prevent mechanical abrasion and scratches. The photosensitive adhesive solidification often accompanies heat release that may cause some side effects to the tissue. In this work, we solidify the skull clearing maintenance reagent by combining the periodic and continuous irradiation, during which the temperature rise is less than 1 °C (Figure S9, Supporting Information), whereas continuous irradiation normally induces over 19 °C rise maximumly (Figure S10, Supporting Information). Previous studies by Kang et al. reported that the application of transparent vinyl polysiloxane (VPS) to the teeth could significantly improve the OCT imaging depth and signal intensity,^[^ ^28^, ^29^ ^]^ which had good biocompatibility.^[^ ^30^ ^]^ Thus, it has a great potential in maintaining skull transparency chronically. Additionally, repeated anesthesia is not conducive to long‐term monitoring of the brain, which may even lead to animal death. The profound anesthetic pharmacologic effects on neural function can confuse the interpretation of experimental research.^[^ ^31^ ^]^ Here using LCCW with a single operation, we can perform imaging in awake mice with the customer‐built holding device and skull holder (Figure S1, Supporting Information).

During the preparation of LCCW, the in vivo skull optical clearing agent is applied, whose effects on skull structure and function are assessed in our recent study.^[^ ^32^ ^]^ Results show that the reagent can lead to the collagen rearrangement, decalcification and lipid dissolution, contributing to the reagent permeability. To assess the penetration of the reagent, we detect the dye penetration in interparietal bone after skull optical clearing treatment. We find that the dye only penetrates into the superficial compact bone as shown in Figure S11A,B (Supporting Information), which is consistent with our recent report.^[^ ^32^ ^]^ Further, we monitor the activation of monocytes in marrow cavity via LCCW within 24 h (Figure S11C, Supporting Information). It can be found that LCCW do not affect cranial bone marrow microenvironment. In addition, we verify the performance of LCCW on both BALB/c and C57BL/6 mice. Results show that LCCW is well performed in both strains of mice for improving OCT imaging quality (Figure S12A, Supporting Information). Moreover, the skull transmittances are very similar for both BALB/c and C57BL/6 mice after applying LCCW (Figure S12B, Supporting Information). Although the skull transmittance is greatly improved by LCCW, there still exists some light attenuation. Specifically, when a low‐power laser (2 mW) is used to induce PT, the transmitted power to the cortex is about 1.6 mW.

Facilitated by LCCW, we monitor the spatiotemporal evolution of vascular remodeling in the cortex and the skull after ischemic injury. The results show that the vascular structure remains stable in normal adult mice (Figure 4). With PT stimuli, the cortical and calvarial blood vessels are disrupted. The cerebral vascular perfusion nearly recovers by the sixth day, which is earlier than that in the previous report.^[^ ^33^ ^]^ The slow recovery is most likely because of the brain immunity deficiency induced by craniotomy used in the previous study, since the mouse meninges contain a pool of myeloid cells and neutrophils supplied by the adjacent skull bone marrow, and there exists the channels connecting the skull bone marrow with the dura for immune cells migration during stroke, according to Cugurra et al.^[^ ^4^ ^]^

In the previous cranial bone marrow studies, researchers usually select bone sutures as the imaging sites, ignoring the interparietal bone.^[^ ^34^, ^35^ ^]^ However, we see that the interparietal bone has more abundant vascular endothelial cells and immune cells than the parietal bone, as shown in Figure S2C,D (Supporting Information). Moreover, the interparietal bone has a regular shape and a large area than the bone sutures, which makes it serve as a more suitable detective target in skull bone marrow study. The two‐photon imaging presents the structure of bone marrow cavities in interparietal bone, which contains plenty of blood vessels and monocytes (Figure S2B, Supporting Information). This unique immune microenvironment may be responsible for the rapid recovery of blood vessels after injury.

Our transcriptome analysis results show that when the brain and the skull are challenged by ischemic injury, their molecular mechanisms show great differences during the recovery process. On the second day after injury, most of genes associated with inflammation significantly upregulate in the brain, similar to the results in previously reported endothelial transcriptome analysis.^[^ ^36^ ^]^ However, the skull shows a completely different response to ischemic injury, since most of the upregulated genes are involved in cell division and cell cycle in the skull. Recruitment of the inflammatory infiltrate can support angiogenesis and tissue remodeling. A recent study reports that macrophages are essential for the repair of the ischemic tissue, and it is consistent with our results of macrophages changes.^[^ ^37^ ^]^ After vascular injury, extensive tissue remodeling occurs, primarily involved in pathways related to immune response, cell adhesion, cell activation, extracellular matrix, proliferation, and migration,^[^ ^38^ ^]^ again consistent with our findings. Surprisingly, most of DEGs associated with neural activity become significantly downregulated for cranial ischemic injury. This means that skull is not only an important part of the brain's special immunity but may also play a key role in maintaining brain function. It is reported that cranioplasty would improve the brain cognitive function over a long period.^[^ ^39^ ^]^ Together with our findings from transcriptome, we believe the skull is much more important to the brain than we ever thought. A concrete understanding of the relationship between skull and brain is to be explored in the future.

## 4 Conclusion

In this study, we propose a novel long‐term optical clearing skull window named LCCW for cortical and calvarial imaging through the intact skull. This method not only enhances the imaging resolution and depth but also allows the long‐term tracing of vascular dynamic changes. We find that calvarial blood vessels recover earlier than the cortex after ischemic injury because of the differences in gene expression patterns and immune cells abundances between them. These findings reveal the vascular injury and regeneration patterns of the cortex and the skull, providing a new hint for clinical treatment of stroke in the future.

## 5 Experimental Section

### Animals

All experimental procedures were performed according to animal experiment guidelines of the Experimental Animal Management Ordinance of Guangdong Province, P. R. China, and the guidelines from the Guangdong Medical University, which were approved by the Institutional Animal Ethics Committee of Central People's Hospital of Zhanjiang (GDY2002085). *Thy1‐YFP‐H*, and *Cx3cr1^EGFP/+^* transgenic mice (The genetic background: C57BL/6) were purchased from Jarvis Bio‐pharmaceutical Co., Ltd. (Wuhan, China), which were applied for neuron and microglia/monocytes imaging, respectively. Otherwise, the wide type male BALB/c mice were used for OCT imaging and ischemic injury modeling. The mice were fed under the specified pathogen free (SPF) condition, with 12 h/12 h light and dark cycle.

### Preparation of Skull Optical Clearing Agent and Skull Clearing Maintenance Reagent

The skull optical clearing solutions consist of S_1_ and S_2_. The S_1_ is the saturated supernatant solution of 75% ethanol and urea at room temperature, and S_2_ is high‐concentration sodium dodecylbenzenesulfonate (SDBS) solution. To prepare the S_1_, 75% ethanol (Sinopharm, China) was mixed with urea (Sinopharm, China), and the mixture should be stirred constantly, then let it stand and remove the supernatant for usage. The volume‐to‐mass ratio of ethanol and urea is about 10:3. As for the S_2_, 0.7 m NaOH solution is mixed with dodecylbenzenesulfonic acid (DDBSA, Aladdin, China) at a volume‐to‐mass ratio of 24:5, and the pH is 7.2–8.0.

Except for skull optical clearing agent, the preparation of LCCW needs the specific skull clearing maintenance reagent to preserve the skull transparency, including primer gel (Superior mixing clear gel, RyujiandMay, China), joint gel (K‐303, Kafuter, China), and sealing gel (plated crystal antifouling sealing gel, Rainey, China). The primer gel mainly consists of acrylic copolymer, polydimethylsiloxane, trimethylolpropane triacrylate, and polymethyl methacrylate. The joint gel is composed of acrylic (ester) prepolymer, acrylate, and photoinitiator. The sealing gel is made of polyacrylate, trimethylolpropane triacrylate, polyurethane‐10, and photoinitiator. The LED light source (365 nm, 5 W) was used to make the gel fully solidify. For establishing a LCCW (5 mm diameter), the doses of primer gel, joint gel, and sealing gel are about 2 µL, 20 µL, and 2 µL, respectively.

### Animal Preparation

The head of anesthetized mouse was shaved, and the scalp was cut off. Then the mucosa on the skull was removed and the skull surface was dried by using clean compressed air. 0.25 g denture base material (Shanghai New Century Dental Materials Co., Ltd., China) and 0.15 mL glue (5800, ergo, Switzerland) were mixed uniformly, then the mixture was applied on the customer‐made holder and adhered with the skull. When the glue mixture was fully dry (about 15 min), the LCCW can be established subsequently. What needs to be emphasized is that the mucosa would tightly adhere to the interparietal bone, thus, it was better to shave off the mucosa slightly by a microblade before the LCCW establishment.

### PT Ischemic Injury Model

With the help of LCCW, focal PT ischemic injury model was allowed to be implemented in male eight‐week‐old BALB/c mice. 100 µL Rose Bengal (RB) solution (4 mg mL^−1^, Sigma‐Aldrich, USA) was injected via the tail vein before the laser irradiation, and the occlusion was induced by 532 nm laser (1 mm diameter, 2 mW, Laserwave, China) irradiation for 210 s.

### OCT Angiography for Cerebral and Calvarial Vascular Imaging

In this work, a commercial spectral domain OCT system (TEL221PSC1, Thorlabs, USA) was used in imaging of microvessels. The light source has a central wavelength of 1300 nm and a full width at half maximum bandwidth of 100 nm. An objective (OCT‐LK3, 5×) lens was used for probing light beam on the region of interest (ROI), which theoretically offers a high axial resolution of 5.5 µm in air and a lateral resolution of 13 µm. The mode of speckle variance angiography was used to obtain the cerebral and calvarial vascular structure, and the areas of ROI are 3.5 cm × 3.5 cm for cerebral vessels and 4.0 cm × 2.5 cm for calvarial blood vessel, respectively. And, the software ThorImageOCT (version 5.4) was used for image acquisition.

### Repeated Imaging in Awake Mice

With the application of LCCW, the repeated imaging in awake mice can be realized. Here, a mouse holding device was designed, as shown in Figure 1A and Figure S1 (Supporting Information). The body of free‐moving mouse was bundled by a thin elastic fabric and then put into the tube. After that, the skull holder was tightened by two screws with the mouse holding device. For the normal mice, the cerebral and calvarial blood vessels were monitored in the first 2 d, and each week for two months. For the PT mice, the imaging time points are the initial, PT‐0 min, 5 min, 10 min, 20 min, 30 min, and 4 h, each day in the first 14 d, as well as 17th, 21th day. To prevent the cranial window from mechanical abrasion and scratches, the LCCW needs to be sealed with a small piece of tape during non‐imaging periods.

### In Vivo Two‐Photon Imaging

Ten‐week‐old male *thy1‐YFP‐H* line mice were used in imaging of neurons to assess the imaging ability achieved by LCCW, with the application of a two‐photon microscope (A1+, Nikon, Japan) coupled a Ti:sapphire laser (Chameleon Vision II, Coherent, USA). Image stacks were obtained with a step size of 2 µm. Imaging parameters were set as: raw images: 512 × 512 pixels, 0.24 µm pixel^−1^; Z‐step: 0.45 µm, excitation: 960 nm, detection: 525 ± 25 nm, objective: 25× W, 1.1 NA, zoom factor: 4. Additionally, the eight‐week‐old male *Cx3cr1^EGFP/+^* mice were used to in vivo monitor the microglia/monocytes activation both in cerebral cortex and skull marrow cavity. After the LCCW establishment, 100 µL tetramethylrhodamine‐conjugated dextran (3 mg mL^−1^, 70 kDa, SigmaAldrich, USA) was injected through the tail vein to label the vessels. The acquisition parameters were set as: raw images: 1024 × 1024 pixels, 0.41 µm pixel^−1^; excitation: 920 nm, detection: 525 ± 25 nm; 575 ± 12.5 nm; 492 SP, objective: 25×W, 1.1 NA. To evaluate the optical aberration, imaging parameters of fluorescent bead were set as: raw images: 2048 × 2048 pixels, 0.05 µm pixel^−1^; excitation: 488 nm, detection: 595 ± 25 nm, objective: 25×W, 1.1 NA, zoom factor: 10. As for assessing the penetration of skull optical clearing agent, the rhodamine (average mol wt 155 000, Sigma‐Aldrich, USA) blended skull optical clearing solutions S_1_ and S_2_ (1 mg mL^−1^) were used to treat the skull. And then the distribution of dye molecules in the skull was monitored. The NIS software controls the instrumentation during acquisition, processing, and storage of image data sets.

### Temperature Record to the Gel Solidification Process

Due to the possible brain injuries caused by the heat release during the process of gel solidification, the thermal infrared imager (TP76, Hikvision, China) was used to measure the temperature changes. The mixture of primer gel and joint gel (G‐1) was added on a slide, and a 5 W LED light source irradiates it at the distance of 30 cm. After that, a thin layer of sealing gel (G‐2) was added on it, and irradiated again. Here, two patterns of the laser irradiation were tested. One is continuous irradiation for 3 min in each step, the other is intermittent irradiation with the cycle of 3 s irradiation and 3 s nonirradiation for 1 min plus continuous irradiation for another 2 min in the first step, as well as the same periodic irradiation manner for 1 min plus 1 min continuous irradiation in the second step.

### Transmittance of Solid Skull Clearing Maintenance Reagent

Here, the spectrophotometer (Lambda1050+, PerkinElmer, USA) was used to measure the transmittance of the solid skull clearing maintenance reagent and skull samples. First, a blank cover glass was placed to measure the transmittance baseline. Then the G‐1 and G‐2 were successively added on the cover glass and solidified, and the transmittance was measured within the range of 300–1500 nm (step size: 5 nm). Additionally, the skull samples from eight‐week‐old male BALB/c and C57BL/6 mice were harvested, and half of them were treated by the skull optical clearing agent and skull transparency maintenance reagent. Then their transmittances were measured by the aforementioned spectrophotometer from 300 to 1500 nm (step size: 5 nm).

### Profiling the Transcriptome in PT Ischemic Injury Models

Here, the transcriptome changes in cortex and skull at representative time points after PT were analyzed. The ischemic and peri‐ischemic tissue was harvested for RNA sequencing.

### RNA‐Sequencing Library Preparation

Total RNA was extracted using TRIzol Reagent (Thermo Fisher Scientific Inc.) and further treated with DNase to remove genomic DNA contamination. The mRNA was isolated using NEBNext Poly(A) mRNA Magnetic Isolation Module (New England Biolabs, Ipswich, MA), and was then used for RNA‐sequencing library preparation with the NEBNext Ultra II mRNA Library Prep Kit for Illumina (New England Biolabs, Ipswich, MA). The library was performed using Illumina sequencing with the paired‐end 2 × 150 sequencing mode.

### Quality Control and Alignment of Sequencing Data

Raw data (raw reads) of fastq format were processed with Fastp (v0.23.0),^[^ ^40^ ^]^ an ultrafast FASTQ preprocessor with useful quality. The clean reads were mapped to the mus musculus genome (mm10) using the HISAT2 software (v2.1.0).^[^ ^41^ ^]^ Differential expression analysis of two groups (two biological replicates per condition) was performed using the DESeq R package (1.18.1).^[^ ^42^ ^]^ The |log_2_(fold change)| > 1 and false discovery rate (FDR) < 0.05 are regarded as differentially expressed genes (DEG).

### Gene Set Enrichment Analysis

The gene set of GO term enrichment and GSEA^[^ ^43^ ^]^ between samples were performed by R‐package clusterProfiler (v4.0)^[^ ^44^ ^]^ with default parameters on the MsigDB C5.^[^ ^45^ ^]^ The heatmap was obtained by the ComplexHeatmap^[^ ^46^ ^]^ with row clustered by Pearson correlation.

### Estimation of Immune Cell Abundance

The immune cell abundances were estimated through expression count data of each sample by ImmuCellAI‐mouse.^[^ ^47^ ^]^ The significant differences of immune cell abundance between injured group and control group were analyzed by two‐sided Student's *t*‐test. The *P‐*values less than 0.05 are considered as significant differences.

### Flow Cytometry for the Skull

The fresh parietal bone and interparietal bone were harvested from eight‐week‐old BALB/c mice. Cell suspension was prepared by mincing the bone, which was then filtered through a 70‐µm cell strainer. And the blood red cells were removed using red cell lysing reagent. After centrifugation at 300 *g* for 5 min at 4 °C, samples were resuspended in 400 µL buffer and kept at 4 °C. Antibodies used in flow cytometry include PE Anti‐Mouse CD31, FITC Anti‐Mouse CD4, and PE Anti‐Mouse CD45 (Proteintech Group, USA).

### Image Processing and Statistical Analysis

In this work, the raw data from OCT angiographic images were processed by using ImageJ for vascular reconstruction. The definition of CNR is defined as follows^[^ ^48^ ^]^

(1) $$CNR=\left(I_{\mathrm{back}}-I_{\mathrm{vessel}}\right)/\sqrt{f_{\mathrm{vessel}}\sigma_{\mathrm{vessel}}^{2}+f_{\mathrm{back}}\sigma_{\mathrm{back}}^{2}}$$

where *I*_vessel_ and *I*_back_ denote the mean values of intensity for vascular area and background, respectively.$\sigma_{\mathrm{vessel}}$and $\sigma_{\mathrm{back}}$ are the corresponding variance in the values. *f*_vessel_ and *f*_back_ denote the fractions of the total number of pixels selected in the image classified as vessels and background, respectively.

Various vessel morphometric and spatial parameters, such as vessel length, vessel density, branching index, and lacunarity, were obtained by AngioTool vascular analysis software.^[^ ^49^ ^]^ Moreover, fluorescence image processing including denoise and 3D reconstruction was achieved by the NIS‐Elements AR analysis software.

### Statistical Analysis

The data were presented as the mean ± standard deviation (SD), which were analyzed by using MATLAB (2016a), GraphPad Prism (Version 8), and R‐package ggplot. The sample sizes are included in the figure legends. The normality of data distribution in each group was checked by Shapiro–Wilk test, and the variance homogeneity was evaluated by Levene's test. The *p*‐values were calculated using one‐way ANOVA, followed by the two‐tailed *t*‐test for data comparison. In this work, *p*‐value < 0.05 is considered a significant difference (n.s.: no significance; *p < 0.05; **p < 0.01; ***p < 0.001).

## Conflict of Interest

The authors declare no conflict of interest.

## Supporting information

Supporting Information

Supplemental Movie 1

Supplemental Movie 2

## Data Availability

The data that support the findings of this study are openly available in Gene Expression Omnibus at https://www.ncbi.nlm.nih.gov/geo/, reference number 191183.
