# Supplementary material for: A Long‐Term Clearing Cranial Window for Longitudinal Imaging of Cortical and Calvarial Ischemic Injury through the Intact Skull
Source: Adv Sci (Weinh). 2022 Apr 9;9(17):2105893. doi: 10.1002/advs.202105893 (PMC9189679; doi:10.1002/advs.202105893)
Supplement: Supplementary file 1 — Supporting Information [file ADVS-9-2105893-s003.pdf]

ADVANCED SCIENCE
Open Access

Supporting Information

for *Adv. Sci.*, DOI 10.1002/advs.202105893

A Long-Term Clearing Cranial Window for Longitudinal Imaging of Cortical and Calvarial Ischemic Injury through the Intact Skull

*Chao Zhang, Chun-Jie Liu and Wei Feng**

Supporting Information

A long-term clearing cranial window for longitudinal imaging of cortical and calvarial ischemic injury through the intact skull

*Chao Zhang, Chun-Jie Liu, Wei Feng**

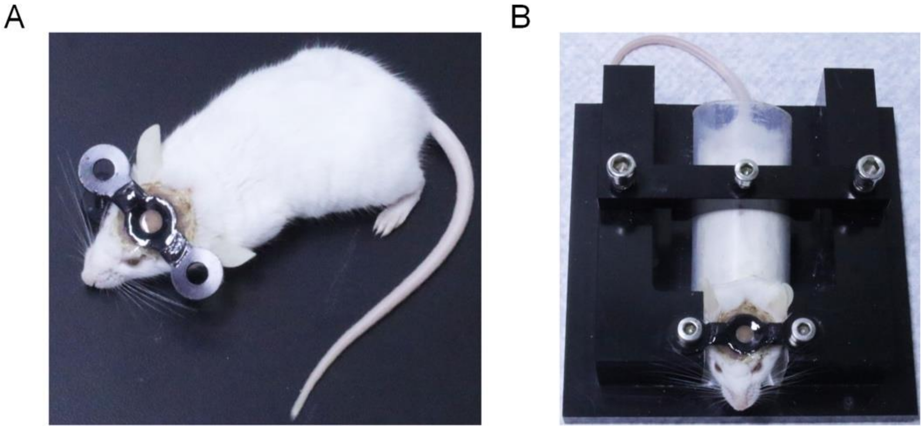

Figure S1. Typical photograph of mouse with LCCW. (A) Representative photograph of an 8-week-old mouse carrying LCCW over the intact skull. (B) Holding device for imaging awake mouse.

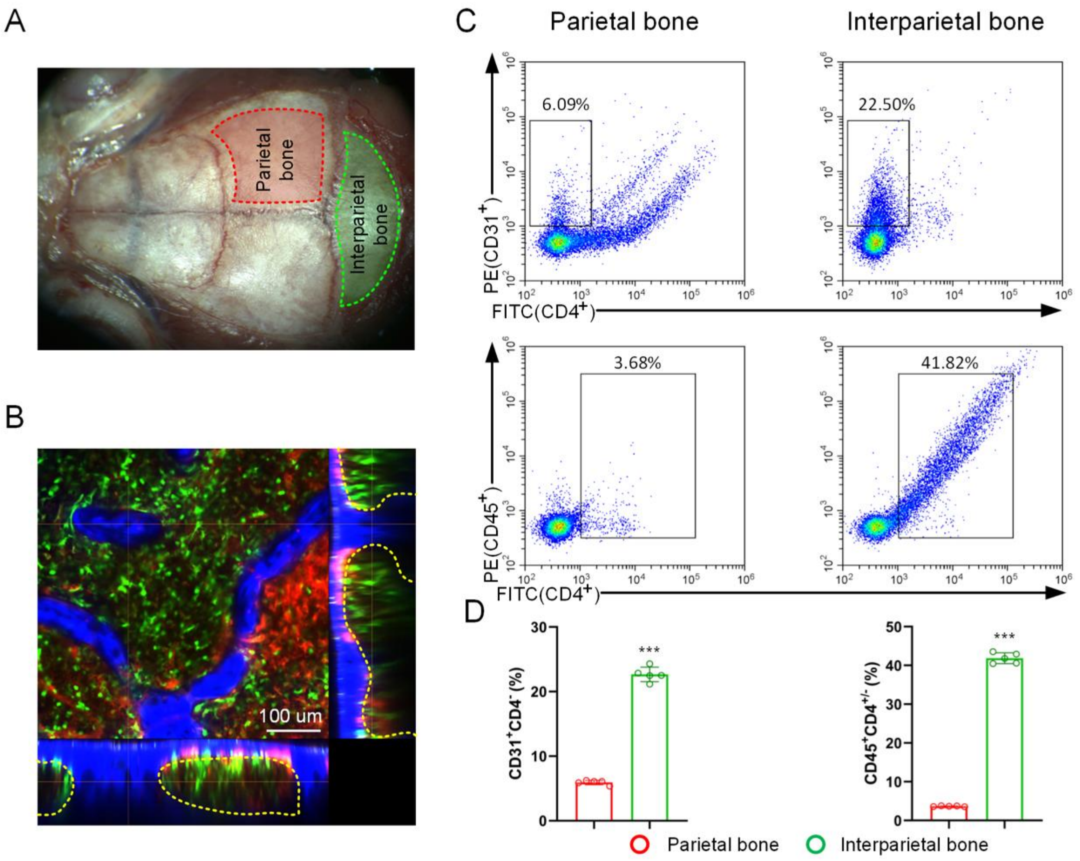

Figure S2. The comparison of the parietal bone and interparietal bone. (A) Photograph of the skull. (B) Image of bone marrow cavities in interparietal bone using *Cx3cr1* $^{EGFP/+}$ mouse. The dashed outline presents the marrow cavity (Green: monocytes; Red: vessel; Blue: skull SHG). (C) Representative flow cytometric graphs of the parietal bone and interparietal bone. (D) Vascular endothelial cell ($CD31^{+}CD4^{-}$) and immune cell ($CD45^{+}CD4^{+/-}$) numbers in skull (n=5, mean ± SD. ***:< 0.001).

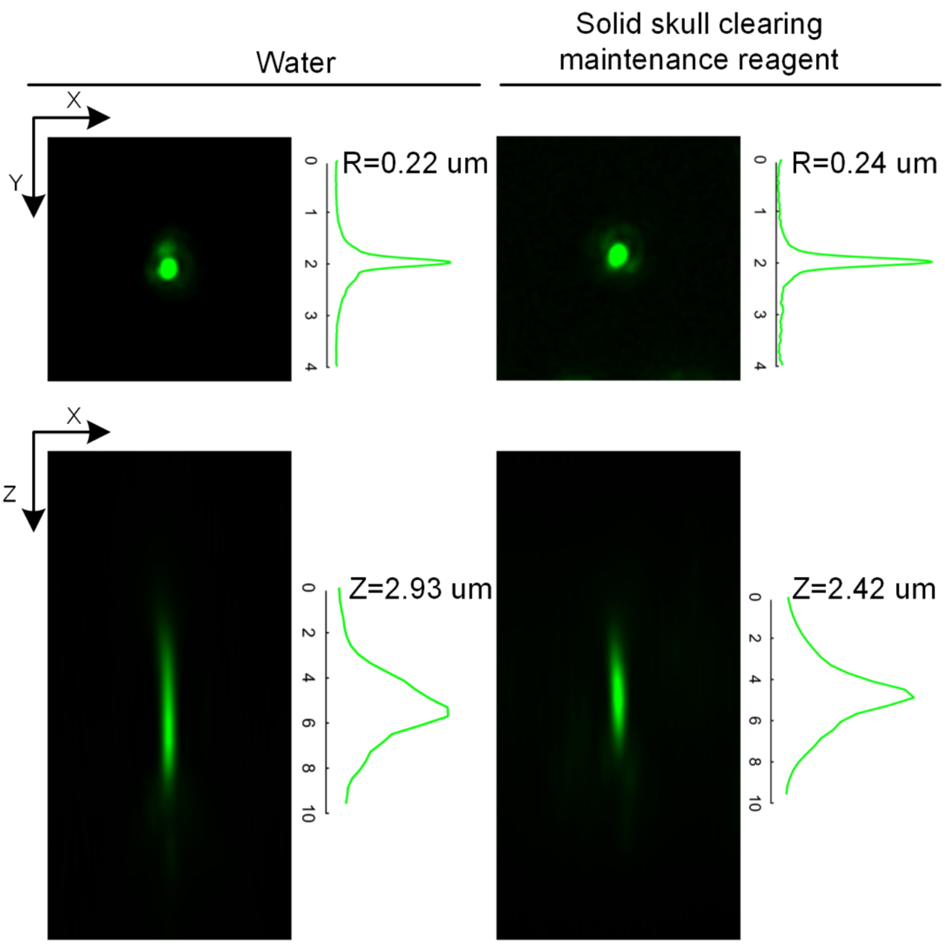

Figure S3. The evaluation of optical aberration in microscopic imaging by the solid skull clearing maintenance reagent. The typical images of first row show the MIP (x-y) and radial depth profile of the integrated axial intensity along the radial axis, respectively. The second row is the MIP (x-z) and axial depth profile of the integrated axial intensity along the z-axis, respectively. The first column is the fluorescent bead imaged by using water dipping objective. The second column is a fluorescent bead imaged under solid skull clearing maintenance reagent with water dipping objective. The R and Z are the full widths that encompass half of the integrated intensity. Raw images are MIP across 0 -28 µm, and the z-step is 0.4 µm.

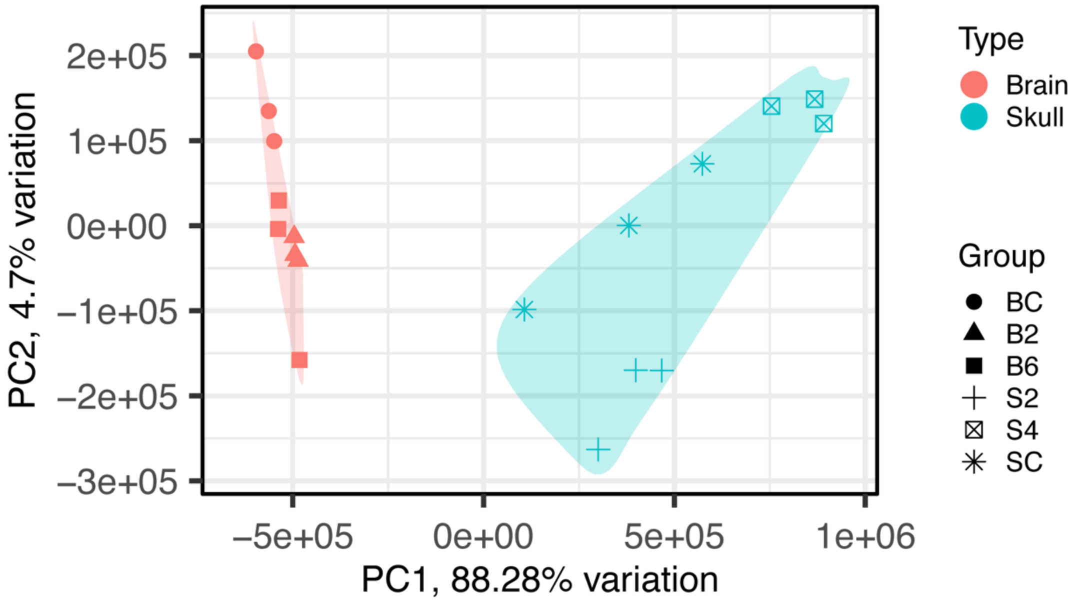

Figure S4. PCA of brain and skull for different timepoints of ischemic injury, analyzed by RNA-seq. (BC: control group for uninjuried brain; B2/6: brain group for the 2/6 day after ischemic injury; SC: control group for uninjuried skull; S2/4: skull group for the 2/6 day after ischemic injury).

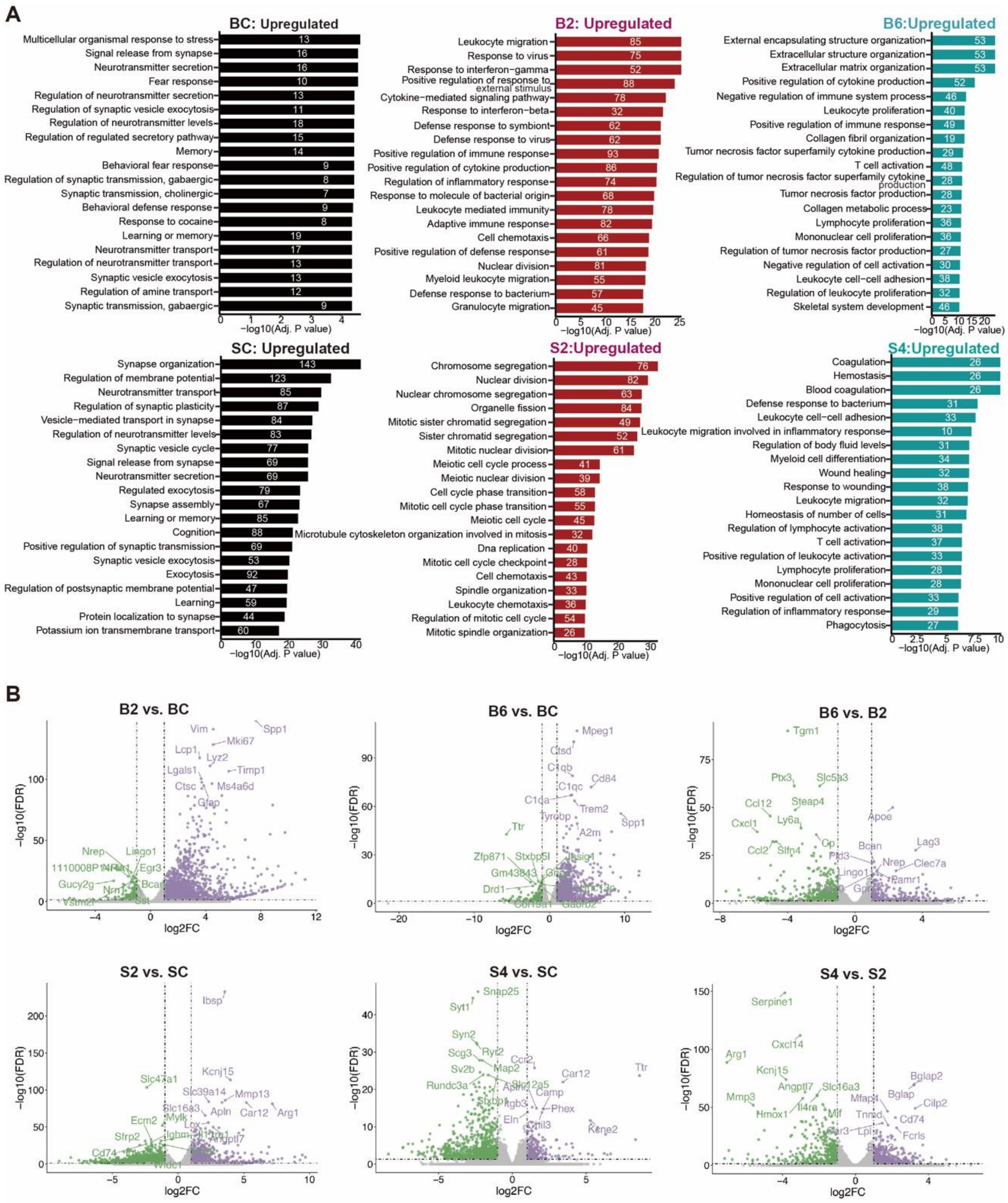

Figure S5. Transcriptome analysis for brain and skull after ischemic injury. (A) GO term of biological process enrichment with up-regulated genes in each group (Top 20 of terms). (B) Volcona plot DEGs for the cortical and calvarial ischemic injury (up-regulated in purple, down-regulated in green). (BC: control group for uninjuried brain; B2/6: brain group for the 2/6 day after ischemic injury; SC: control group for uninjuried skull; S2/4: skull group for the 2/4 day after ischemic injury).

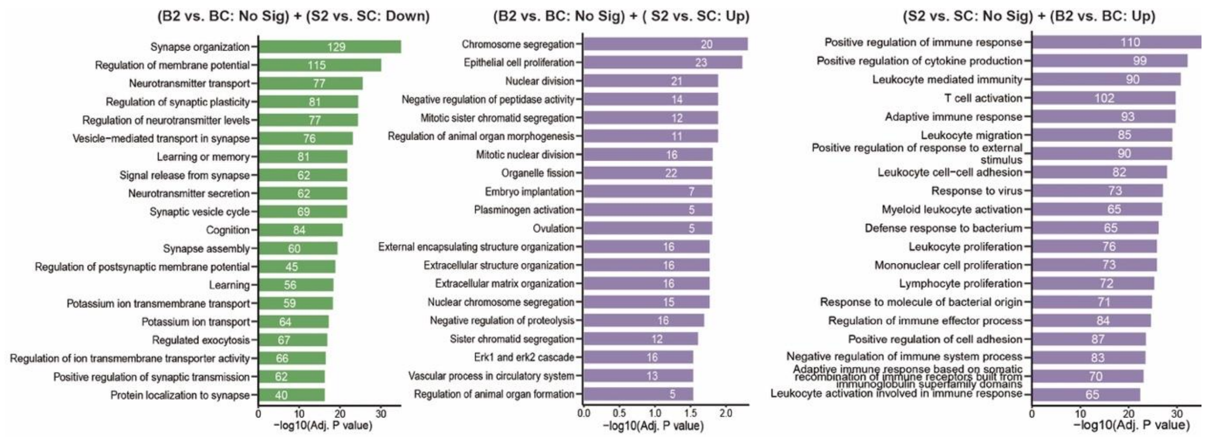

Figure S6. GO term of biological process enrichment with interesected genes between B2 *vs.* BC and S2 *vs*. SC. (up-regulated in purple, down-regulated in green). (BC: control group for uninjuried brain; B2: brain group for the 2nd day after ischemic injury; SC: control group for uninjuried skull; S2: skull group for the 2nd day after ischemic injury).

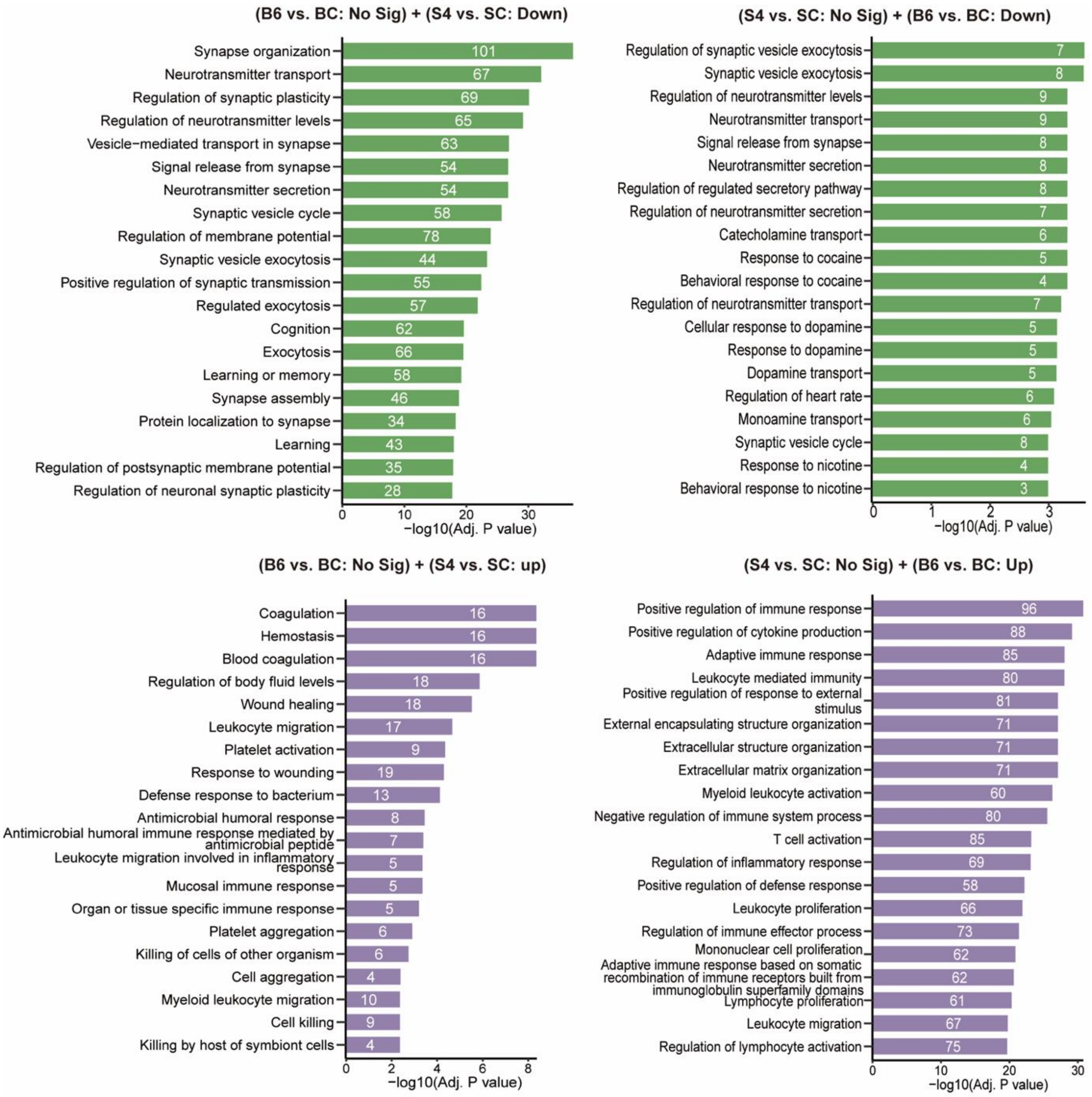

Figure S7. GO term of biological process enrichment with interesected genes between B6 vs BC and S4 vs. SC (up-regulated in purple, down-regulated in green). (BC: control group for uninjuried brain; B6: brain group for the 6th day after ischemic injury; SC: control group for uninjuried skull; S4: skull group for the 6th day after ischemic injury).

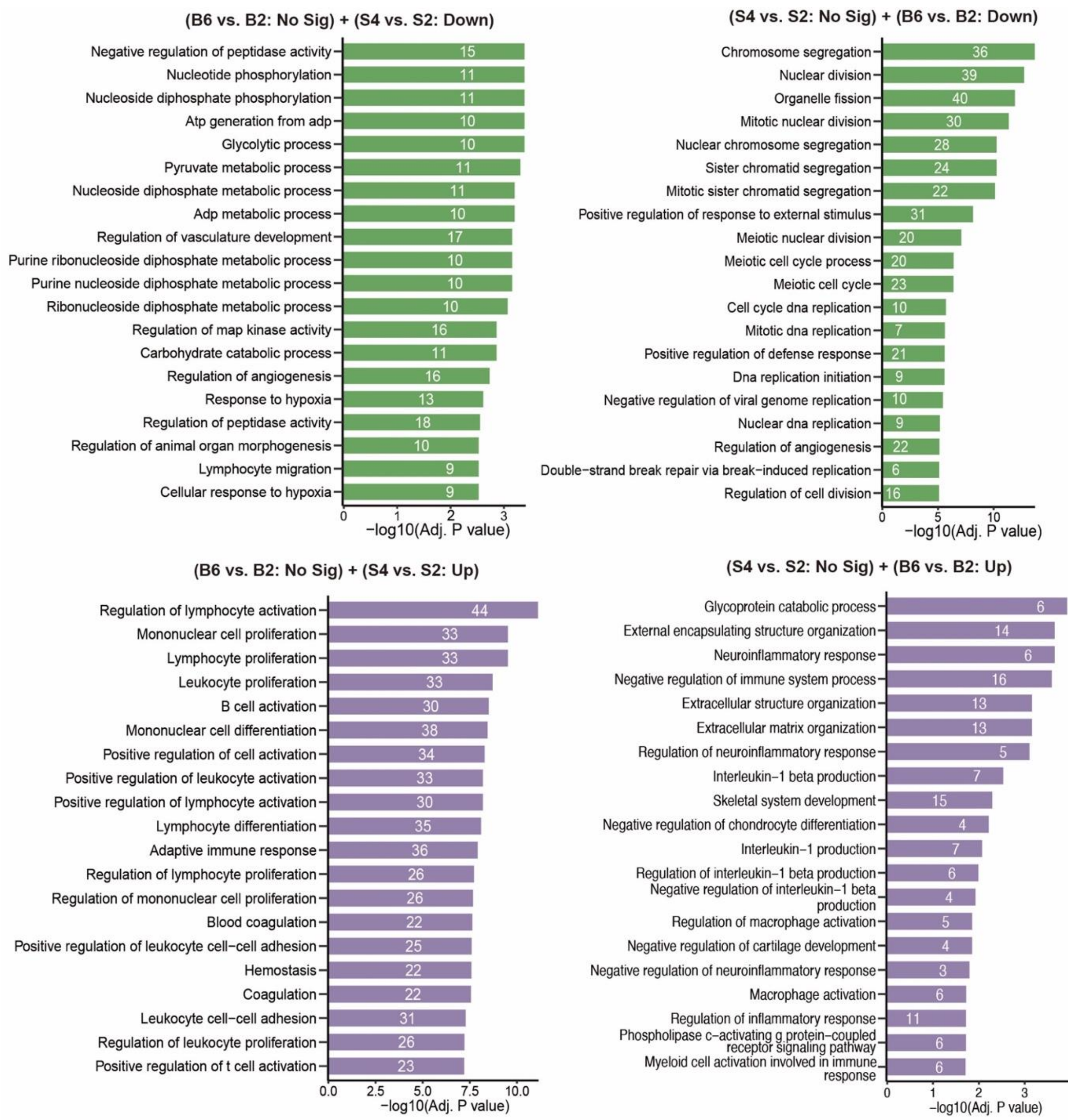

Figure S8. GO term of biological process enrichment with interesected genes between B6 vs B2 and S4 vs. S2 (up-regulated in purple, down-regulated in green). (BC: control group for uninjuried brain; B2/6: brain group for the 2/6 day after ischemic injury; SC: control group for uninjuried skull; S2/4: skull group for the 2/4 day after ischemic injury).

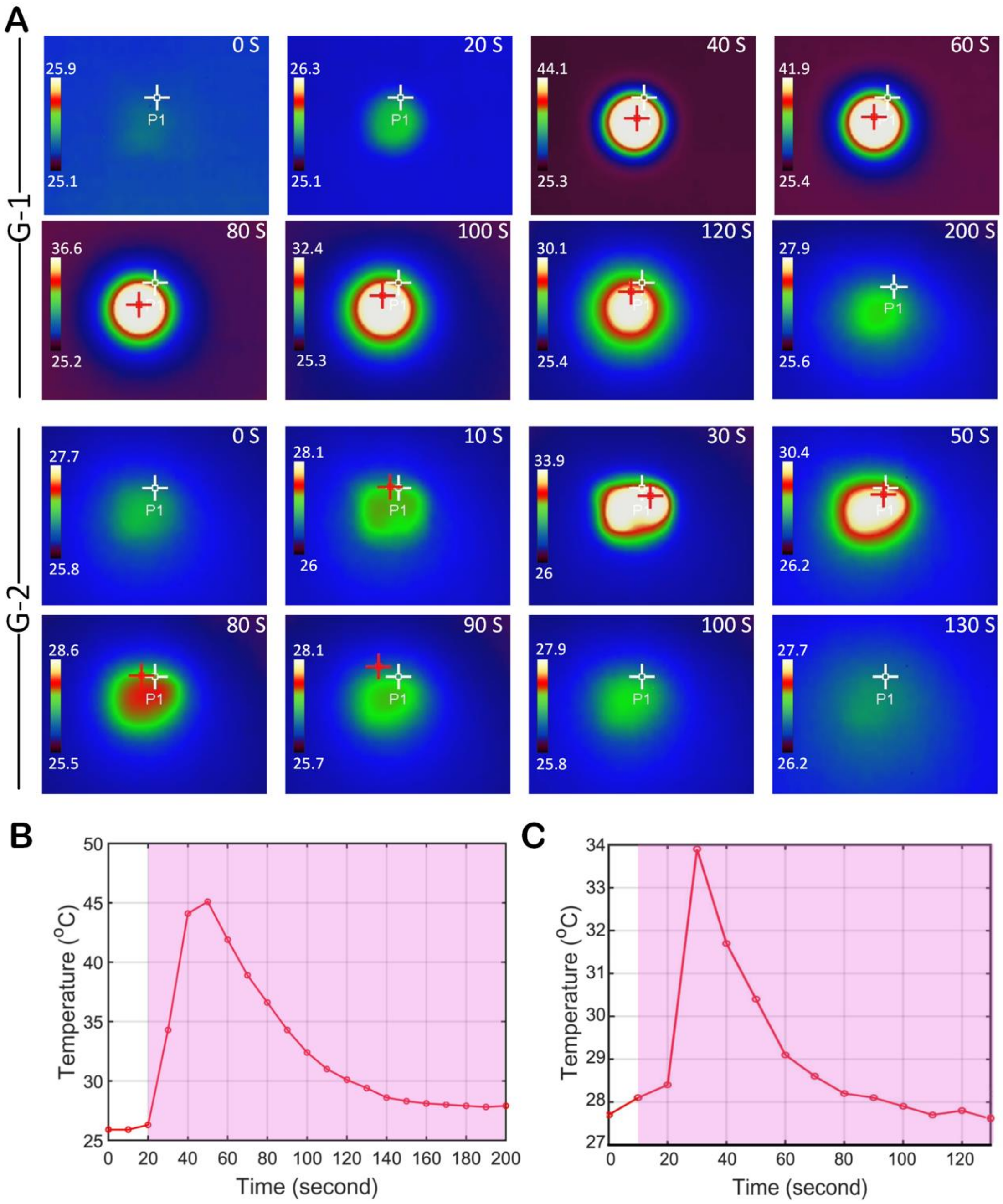

Figure S9. The temperature records during adhesive solidification with continuous light irradiation. (A) Infrared thermal images of G-1 (the mixture of primer gel and joint gel) and G-2 (sealing gel) before and after light irradiation. Regional maximum temperature change of G-1(B) and G-2 (C). (Pink shade: continuous irradiation).

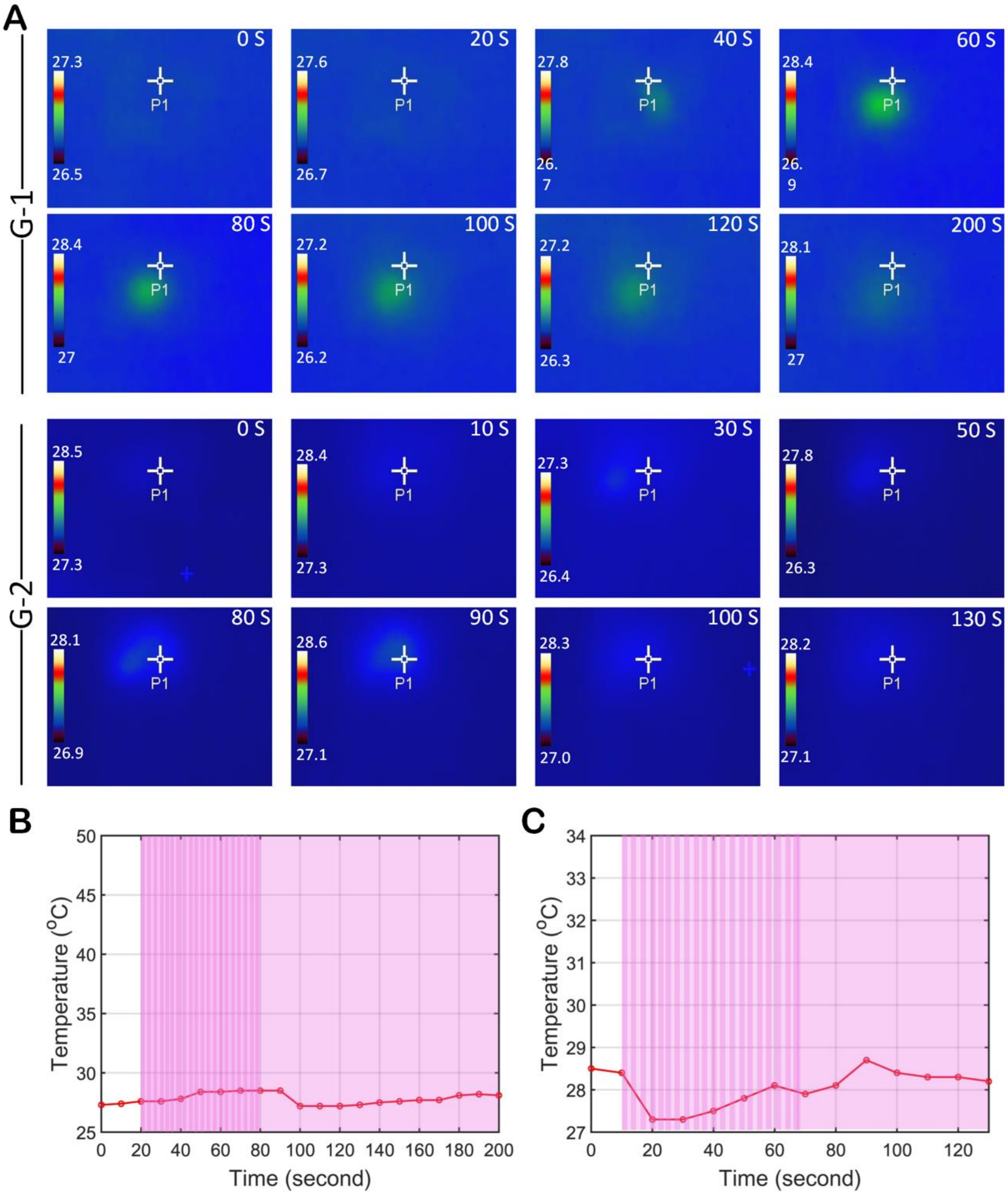

Figure S10. The temperature records during adhesive solidification with intermittent plus continuous light irradiation. (A) Infrared thermal images of G-1 (the mixture of primer gel and joint gel) and G-2 (sealing gel) before and after light irradiation. Regional maximum temperature change of G-1(B) and G-2 (C). (Deep pink stripe: intermittent light irradiation; Pink shade: continuous irradiation).

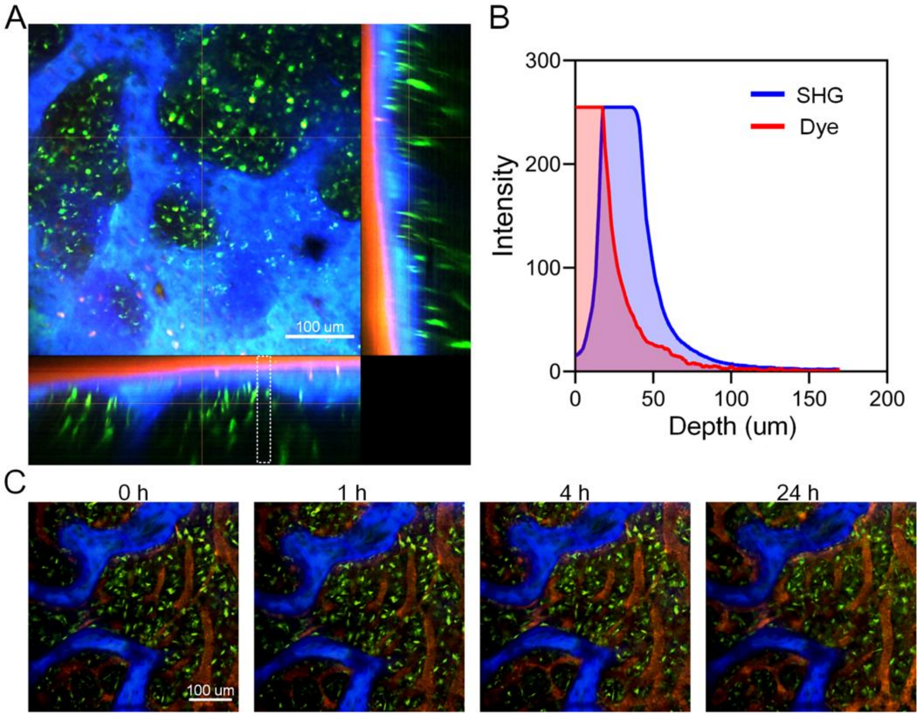

Figure S11. Monitoring of calvarial marrow microenvironment using two-photon imaging. (A) The diffusion of fluorescent dye (Rhodamine) in skull after in vivo skull optical clearing agent treatment (Blue: SHG; Red: dye; Green: monocyte). (B) The profiles of fluorescent dye and SHG intensity maps along the rectangular box in the XZ section in A. (C) Monitoring the activation of monocytes within 24 hours (Green: monocytes; Red: vessel; Blue: skull SHG).

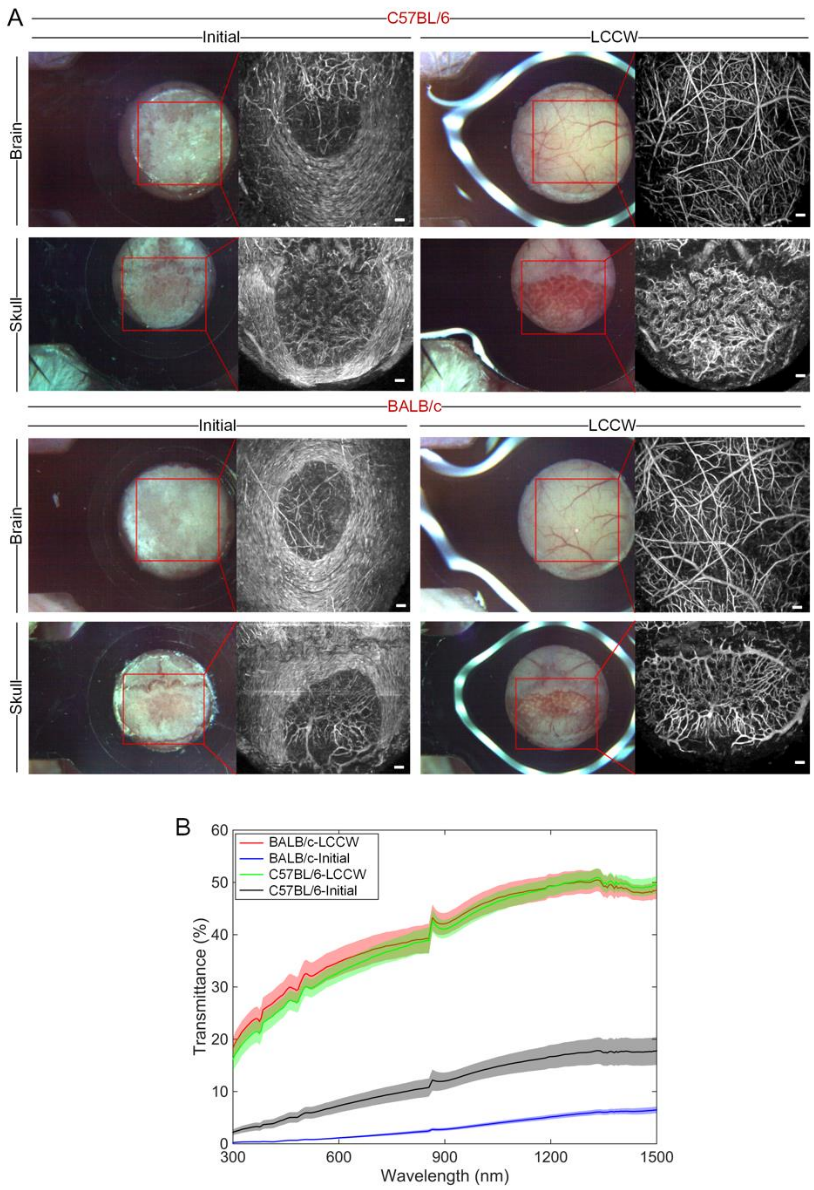

Figure S12. The imaging performance of LCCW in C57BL/6 and BALB/c mice. (A) Imaging blood vessels through dry skull (Initial) and LCCW in 8-week-old C57BL/6 and BALB/c mice. The maps are MIP views of cortical and calvarial vessels in depth direction (from surface to bottom, depth 560 μm; Scale bar 250 μm). (B) Transmittance of skull with or without the LCCW. The curves and the width of shadows are mean and standard deviation, respectively. (n=5, mean ± standard deviation)

Table S1. The number of differently expressed genes (DEG) after ischemic injury in brain and skull. (BC: control group for uninjuried brain; B2/6: brain group for the 2/6 day after ischemic injury; SC: control group for uninjuried skull; S2/4: skull group for the 2/4 day after ischemic injury).

	**B2 vs. BC**	**B6 vs. BC**	**B6 vs. B2**
**Up**	1474	1090	302
**Down**	193	136	508
**DEG**	1667	1226	810
	**S2 vs. SC**	**S4 vs. SC**	**S4 vs. S2**
**Up**	802	268	551
**Down**	1645	871	452
**DEG**	2447	1139	1003
